# Continuous adiabatic frequency conversion for FMCW-LiDAR

**DOI:** 10.1038/s41598-024-55687-1

**Published:** 2024-02-29

**Authors:** Alexander Mrokon, Johanna Oehler, Ingo Breunig

**Affiliations:** 1 Laboratory for Optical Systems, Department of Microsystems Engineering - IMTEK, University of Freiburg, Georges-Köhler-Allee 102, Freiburg, 79110 Germany; 2 Fraunhofer Institute for Physical Measurement Techniques IPM, Georges-Köhler-Allee 301, Freiburg, 79110 Germany

**Keywords:** Adiabatic frequency conversion, Whispering gallery resonators, Electro-optic effect, Lithium niobate, Optical physics, Applied optics, Lasers, LEDs and light sources

## Abstract

Continuous tuning of the frequency of laser light serves as the fundamental basis for a myriad of applications spanning basic scientific research to industrial settings. These applications encompass endeavors such as the detection of gravitational waves, the development of precise optical clocks, environmental monitoring for health and ecological purposes, as well as distance measurement techniques. However, achieving a broad tuning range exceeding 100 GHz along with sub-microsecond tuning times, inherent linearity in tuning, and coherence lengths beyond 10 m presents significant challenges. Here, we demonstrate that electro-optically driven adiabatic frequency converters utilizing high-*Q* microresonators fabricated from lithium niobate possess the capability to convert arbitrary voltage signals into frequency chirps with temporal resolutions below 1 µs. The temporal evolution of the frequency correlates accurately with the applied voltage signal. We have achieved to generate 200-ns-long frequency chirps with deviations of less than 1 % from perfect linearity without requiring supplementary measures. The coefficient of determination is $R^2>0.999$. Moreover, the coherence length of the emitted light exceeds 20 m. To validate these findings, we employ the linear frequency sweeps for Frequency-Modulated Continuous Wave (FMCW) LiDAR covering distances ranging from 0.5 to 10 m. Leveraging the demonstrated nanosecond-level tuning capabilities, coupled with the potential to tune the eigenfrequency of lithium-niobate-based resonators by several hundred GHz, our results show that electro-optically driven adiabatic frequency converters can be used in applications that require ultrafast and flexible continuous frequency tuning characterized by inherent linearity and substantial coherence length.

## Introduction

For over half a century, scientists have been engaged in the pursuit of manipulating the emission frequency of lasers. Continuous (mode-hop free) tuning, in particular, holds significant importance for numerous applications, facilitating the active stabilization of laser light to a desired frequency value and enabling frequency sweeps, both of which are critical across various domains. One such critical application is the detection of gravitational waves, which relies on an interferometer driven by a laser that has its frequency meticulously stabilized^1^. In the field of exoplanet discovery, stabilized frequency combs play a pivotal role^2^. Additionally, the realization of optical clocks involves the cooling and trapping of atoms using laser light with a precisely stabilized frequency^3^. Moreover, health and environmental monitoring heavily depend on high-resolution laser spectroscopy of naturally occurring molecules^4^, while artificial (photonic) molecules can also be thoroughly investigated for various purposes^5^. Determining distances and velocities is accomplished through the utilization of frequency-modulated continuous-wave (FMCW) LiDAR technology^6^. Swept source optical coherence tomography (OCT) finds extensive use in medical imaging^7^. Therefore, the precise control of the frequency of laser light is not only of paramount importance in fundamental scientific research but also holds immense significance in a multitude of practical, real-world applications.

Various approaches exist for achieving continuous tuning of the frequency of laser light, each offering distinct advantages and limitations. The potential domains of application are determined by specific parameters including tuning range, tuning time, tuning linearity, and coherence length. The tuning rate, a measure of the speed of frequency tuning, is derived from the ratio of tuning range to tuning time. The tuning approaches can be classified into two fundamental concepts: the first involves active modulation of the laser source itself, while the second entails frequency conversion of light emitted by a laser without direct manipulation of the light source. Subsequently, we compare the different techniques with respect to the aforementioned parameters.

Distributed Feedback (DFB) lasers, readily available in commercial markets, can be adjusted by modulating the driving current. This method typically yields tuning ranges of several tens of gigahertz within microseconds, resulting in tuning rates around 10 GHz/µs. However, this straightforward tuning mechanism exhibits nonlinearity^8^. The coherence length is approximately 10 meters. Combining 12 DFB elements, researchers managed to achieve a tuning range of 5560 GHz within 3000 µs. By actively implementing linearization techniques for the frequency sweep, this approach was successfully utilized for distance measurements up to 6 meters, achieving resolutions better than 30 micrometers via Frequency-Modulated Continuous-Wave (FMCW) LiDAR^9^.

Moreover, commercially available micro-electro-mechanically tuned vertical-cavity surface emitting laser systems (MEMS-VCSELs) encompass approximately 20 terahertz at 1 µm central wavelength within 2 µs, yielding a tuning rate of $10^4$ GHz/µs. However, achieving tuning linearity requires additional measures, such as employing reference interferometers or programmed controllers^10–13^. The coherence length is contingent upon both the tuning range and the tuning speed, typically falling within the range of 1 m. Nonetheless, constraining the tuning range or utilizing lower tuning rates can enhance the coherence length by up to two orders of magnitude^14^. With effective tuning linearization, MEMS-VCSELs find applications in medical imaging via Optical Coherence Tomography (OCT) or for long-distance measurements through Frequency-Modulated Continuous-Wave (FMCW) LiDAR^15^.

Semiconductor-based akinetic swept sources achieve extensive and linear frequency tuning solely through electronic control^16^. By synchronizing five control voltages, a linear frequency sweep is attained. These lasers offer a tuning range of 20 THz at 1.3 µm central wavelength within microseconds, resulting in a tuning rate of 4000 GHz/µs. Coherence lengths typically reach around 0.1 m. Such lasers find application in optical imaging via Optical Coherence Tomography (OCT)^16,17^.

To achieve linear frequency tuning while maintaining a substantial coherence length spanning hundreds of meters, a semiconductor laser or amplifier can be coupled with an external high-quality-factor resonator. Laser tuning is facilitated by modulating the resonance frequency of the cavity utilizing the linear electro-optic effect (Pockels effect)^18,19^. With this method, a tuning range of 2 GHz is achievable within 1 nanosecond, equivalent to a tuning rate of 2000 GHz/µs^20^. However, compared to the aforementioned techniques, a significant limitation lies in the constrained tuning range, which is directly proportional to the quality factor of the resonator^21^. Consequently, a higher quality factor is advantageous for achieving a broader tuning range. Nonetheless, it has been demonstrated that the tuning time must be significantly shorter than the photon lifetime; therefore, for higher tuning rates, a lower quality factor is required. Consequently, it’s not feasible to simultaneously maximize tuning range and minimize tuning time. However, successful application for FMCW LiDAR was demonstrated^18^.

In summary, none of the aforementioned methods successfully combines a wide tuning range, short tuning times, tuning linearity, and extensive coherence length. Additionally, they are all inherently constrained by the gain bandwidth of the laser medium. The second approach is based on the conversion of the frequency of laser light, with one promising method being adiabatic frequency conversion (AFC). Here, laser light is coupled into an optical resonator. Then, the optical length of the cavity, i.e. its eigenfrequency, is modulated on a time scale smaller than its decay time^22^. Consequently, the light stored within the resonator undergoes frequency modulation corresponding to the changes in eigenfrequency. This process mirrors the optical equivalent of adjusting sound pitch by modifying the length of a guitar string. In an first experimental demonstration, light with a wavelength of 1.5 µm was injected into a silicon ring resonator. A short laser pulse induces mobile charges in the silicon, thereby altering the material’s refractive index and resulting in frequency shifts of 300 GHz within a picosecond, equivalent to a tuning rate of $3\times 10^8$ GHz/µs^23^. Thus, already in its first experimental demonstration, this technique outperforms the others by several orders of magnitude in terms of tuning rate. However, this configuration exhibits limitations: the adiabatic frequency change is proportionate to the energy of the control pulse, posing challenges for achieving a continuous linear frequency sweep. Moreover, it confines frequency shifts to positive values, and employing a pulsed laser to drive the system entails considerable effort.

In order to change this unsatisfactory situation and to unlock the potential of AFC for ultrafast, flexible and continuous frequency tuning of laser light, we have replaced laser-based charge injection with the linear electro-optic effect^24^. This substitution offers a rapid and linear response, along with flexibility in changing the sign of the frequency change. Using this scheme with a millimeter-sized resonator made of lithium niobate, we have achieved 5 GHz tuning range in 5 ns, translating to 1000 GHz/µs tuning rate^25^. Recently, electro-optically driven adiabatic frequency tuning of 14 GHz in 14 ps, i.e. $10^6$ GHz/µs was demonstrated with a chip-integrated microresonator made of lithium niobate^26^.

**Figure 1:**
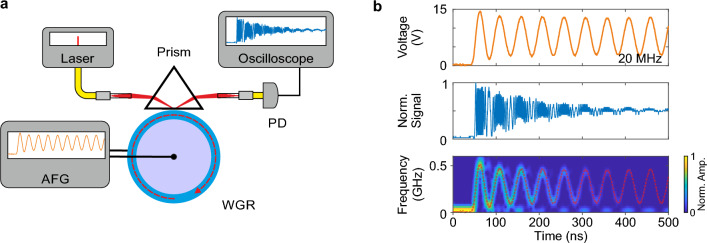
Electro-optically driven adiabatic frequency converter. (**a**) Schematic setup including the pump laser, the coupling prism and the whispering gallery resonator (WGR). An arbitrary function generator (AFG) modulates the eigenfrequency of the cavity and consequently the frequency of the light coupled out of the prism. The beat signal between unconverted light and converted one is detected with a photodiode (PD) and displayed with an oscilloscope. (**b**) Temporal behaviors of a signal driving the AFG with 20 MHz, of the corresponding normalized beat signal and the derived frequency shifts. The dashed red line shows a linearly scaled version of the driving electrical signal.

Given that frequency modulation in this setup relies on the linear electro-optic effect while keeping the laser unaltered, it should theoretically be feasible to generate linear frequency sweeps with coherence lengths exceeding 10 m. Consequently, electro-optically driven adiabatic frequency conversion holds promise for combining ultrafast and versatile frequency tuning with superior linearity and extensive coherence length. However, there is still a need for a thorough investigation of continuous adiabatic frequency tuning, and its application in scenarios necessitating continuous tuning has yet to be realized. Currently, there may be doubts regarding the practical utility of adiabatic frequency converters for real-world applications. In this study, we aim to investigate the continuous tuning capabilities of electro-optically driven adiabatic frequency converters, with a specific emphasis on assessing the linearity of the tuning mechanism. Additionally, we intend to implement this tuning approach for Frequency-Modulated Continuous-Wave (FMCW) LiDAR, which imposes stringent requirements on the characteristics of the light source.

## Results

### Tuning linearity

An electro-optically operated adiabatic frequency converter alters the frequency $\nu$ of laser light as it is coupled into a microresonator of thickness *d* by^24^

1 $$\begin{aligned} \Delta \nu = \frac{1}{2}\nu n^2 r \eta \frac{U}{d}\;. \end{aligned}$$

In this equation, *n* represents the refractive index of the resonator material, *r* signifies its electro-optic coefficient, and *U* stands for the voltage applied across the electrodes located on the top and bottom surfaces of the resonator. The factor $\eta$ accommodates for any deviation in geometry from that of a plate capacitor^27^. Equation (1) illustrates that a voltage signal *U*(*t*) which varies continuously results in a linear conversion to a frequency shift $\Delta \nu (t)$, provided that the variation occurs on a timescale smaller than the resonator’s decay time.

To confirm this, we employ the arrangement sketched in Fig. 1a. Laser light at $\nu =192$ THz frequency (1560 nm wavelength) is coupled into a millimeter-sized whispering gallery resonator made of lithium niobate using a rutile prism. An arbitrary function generator supplies a voltage signal *U*(*t*) that varies over time. The light, shifted in frequency to $\nu +\Delta \nu (t)$, exits the resonator and interferes with the unshifted laser light at $\nu$, generating a beat signal detected by a photodiode linked to an oscilloscope. From the beat signal, we extract the actual frequency change denoted as $\Delta \nu (t)$ and compare its temporal dynamics with that of *U*(*t*).

We start our analysis with a 500-ns duration signal, ranging from 0 to 15 V, comprising a 20 MHz sinusoidal oscillation. Figure 1b illustrates this signal alongside the corresponding beat signal and the frequency shift. The beat signal distinctly exhibits low beat frequencies at low voltages and high beat frequencies at high voltages. Additionally, it indicates that the resonator has a decay time of several 100 ns. The time-dependent behavior of the frequency shift exhibits all characteristics present in the driving voltage signal. The 20 MHz oscillation is clearly evident, along with the overshoot during the initial oscillation, indicating that even minute details of the voltage signal are faithfully reflected in the frequency shift. At 15 V driving signal, we observe a 510 MHz frequency shift.

**Figure 2:**
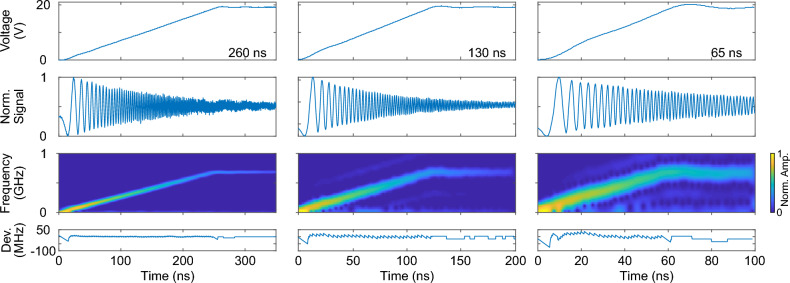
Generation of linear frequency chirps. Driving voltages from the arbitrary function generator, corresponding normalized beat signals, the derived frequency changes and the deviation from a perfect linear frequency chirp. The respective signals are displayed for 260, 130 and 65 ns rise times.

For a more rigorous analysis, we focus on linear frequency chirps as they hold significant relevance for various applications. We configure the arbitrary function generator to produce driving signals where the voltage increases linearly from 0 to 20 V within durations of 260, 130, and 65 ns, respectively. These signals are depicted in Fig. 2 along with the corresponding normalized beat signals, frequency changes, and deviations from ideally linear frequency chirps. With a rise time of 260 ns, the function generator delivers an almost flawless linear voltage increase. The associated frequency change exhibits a consistent rise in frequency from 0 to 690 MHz. Deviations from a perfectly linear chirp remain below 10 MHz for a time interval between 20 and 250 ns. Notably, larger deviations are only observed from 0 to 20 ns and from 250 to 260 ns. Throughout the entire duration, the root mean square (rms) value of the deviation is 5 MHz. A linear regression analysis yields a coefficient of determination, which serves as an indicator of the goodness of fit, with $R^2=0.999$.

Examining frequency chirps with shorter rise times (130 and 65 ns) and contrasting them with those of the 260 ns chirp reveals the following observations: As the rise time decreases, the voltage signal provided by the function generator exhibits a less linear increase from 0 to 20 V. Nevertheless, in all cases, the frequency steadily rises from 0 to 690 MHz. However, both the uncertainty of the frequency change and the deviation from a perfectly linear chirp notably increase as the rise time decreases. For rise times of 130 and 65 ns, we ascertain rms values of 11 and 20 MHz, respectively, along with $R^2$ values of 0.995 and 0.985, respectively. Similar to the 260 ns chirp, the deviation from perfect linearity appears to be significantly larger at the onset of the chirp, specifically for times less than 10 ns.

### Application for FMCW-LiDAR

The results presented above illustrate the potential of adiabatic frequency converters in producing linear frequency chirps. Our next objective is to investigate whether these devices can indeed function as practical light sources. To explore this, we employ an electro-optically driven adiabatic frequency converter to measure distances of up to 10 m using FMCW LiDAR as a proof-of-concept. Here, it is essential to combine linear frequency tuning with a coherence length extending several tens of meters.

**Figure 3:**
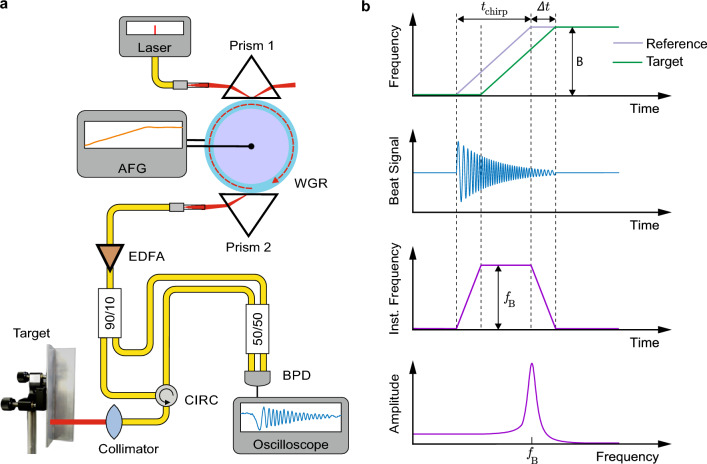
Adiabatic frequency converter for FMCW LiDAR. (**a**) Experimental setup comprising an electro-optically driven adiabatic frequency converter sketched in Fig. 1. The frequency shifted light is coupled out using an additional prism (Prism 2). Optical fiber splitters and a circulator (CIRC) ensure that reference light and light reflected from the distant target form a beat signal at a balanced photodetector (BPD) connected to an oscilloscope. (**b**) Determination of the maximum beat frequency $f_{\textrm{B}}$ which is proportional to the distance of the target. Interfering linearly chirped reference and target signals with the bandwidth *B* form a beat signal with a temporally varying frequency having the maximum value $f_{\textrm{B}}$. Fourier transform of the beat signal directly yields $f_{\textrm{B}}$.

The experimental arrangement is depicted in Fig. 3a. It consists of the previously described adiabatic frequency converter supplemented with a second prism. This addition ensures the coupling out of frequency-chirped light without allowing it to interfere with non-converted light.

The chirp is characterized by the bandwidth *B* and the chirp time $t_{\textrm{chirp}}$, represented by the slope $B/t_{\textrm{chirp}}$. Approximately 10 percent of the output light serves as the reference signal, while 90 percent are directed towards a distant target, specifically a non-polished metal plate. The light reflected from the target and the reference light interfere on a balanced photodiode. These signals exhibit the time delay $\Delta t$, which is proportional to the distance of the target. The time delay is transferred into a beat signal visualized on an oscilloscope. The linearly chirped reference and target signals, along with the resulting beat signal, are illustrated in Fig. 3b. During the time interval $\Delta t$, the frequency of the beat signal linearly increases from 0 to the maximum value $f_{\textrm{B}}=(B/t_{\textrm{chirp}})\Delta t$, remains constant at $f_{\textrm{B}}$ for $t_{\textrm{chirp}}-\Delta t$, and then decreases back to 0 during $\Delta t$ (see to Fig. 3b). We have two options for determining $f_{\textrm{B}}$ and consequently the distance from the target. We can either analyze the temporal behavior of the instantaneous frequency as we did it in the linearity analysis above, or we can Fourier transform the beat signal.

**Figure 4:**
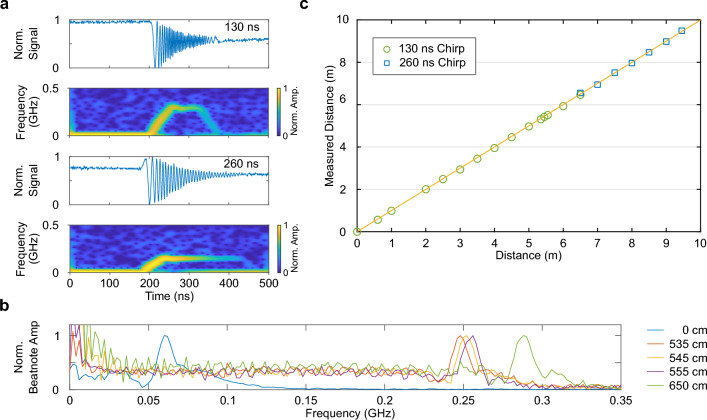
Distance measurement using frequency chirps from an adiabatic frequency converter. (**a**) Beat signals and corresponding temporal evolution of the derived frequency shifts for 650 cm distance with 130 and 260 ns rise time, respectively. (**b**) Normalized Fourier transform of beat signals for different distances between 0 and 650 cm for 130 ns rise time. (**c**) Distance determined with AFC-based FMCW LiDAR (green circles and blue squares) vs. distance measured with a commercially available device based on intensity modulation. The yellow line represents a perfect correlation between the two measurement methods.

Given that the balanced photodiode’s bandwidth is limited to 300 MHz, it is important to ensure that the beat frequency remains below this threshold. Hence, we conduct measurements within the range of 0 to 6.5 m with a chirp duration of $t_{\textrm{chirp}}=130$ ns, and from 6.5 to 10 m with $t_{\textrm{chirp}}=260$ ns. Figure 4a shows the measured beat signals and the corresponding temporal beat-frequency evolutions for both chirp durations at a distance of 6.5 m. The expected temporal behavior sketched in Fig. 3b is nicely reproduced. The only significant difference between the two lies in the magnitude of the maximum frequency. With a rise time of 130 ns, we observe a peak frequency of 290 MHz, while for 260 ns, the maximum frequency is halved. Figure 4b displays the Fourier transform of beat signals for a rise time of 130 ns and distances ranging from 0 to 6.5 m. At zero distance, there is a prominent peak at 60 MHz, shifting to 290 MHz at 6.5 m. The temporal behavior analysis and Fourier transform yield consistent results. The full width at half maximum of the peaks is 10 MHz. To assess resolution, we varied the target distance in 10 cm increments from 5.35 to 5.55 m, resulting in respective frequencies $f_{\textrm{B}}$ of 248, 252, and 255 MHz.

Using the beat frequencies, we can ascertain the distance of the target, noting that 60 MHz corresponds to zero distance. We systematically varied the target’s distance from 0 to 10 m and measured its magnitude utilizing our AFC-based FMCW LiDAR configuration. Figure 4c compares the distance magnitudes determined with the AFC-based light source with those acquired using a commercially available device employing light-intensity modulation. There is no significant deviation between the values.

## Discussion

The experimental findings concerning tuning linearity reveal that a maximum driving signal of 15 V (20 V) results in a maximum frequency shift of 510 MHz (690 MHz). When employing Eq. (1) with $\nu =192$ THz, $n=2.13$^28^, $r=25$ pm/V^29^, $\eta =0.95$^27^, and $d=300$ µm, the experimentally derived values align closely with the expected ones. Notably, the linearity of frequency chirps diminishes as the rise time of the voltage signal decreases. The most significant deviation from linearity occurs within the initial 10 to 20 ns of the chirps, independent of rise time. We attribute this to the frequency shift determination algorithm, which necessitates oscillations both before and after the designated time for frequency shift determination. Consequently, near the onset of voltage increase, the algorithm’s efficacy diminishes. Moreover, the uncertainty associated with the determined frequency shift increases as rise time decreases, due to the algorithm’s requirement for a certain number of oscillations to reliably determine frequency.

Importantly, it is observed that the nonlinearity of the driving voltage signal increases with decreasing rise time, owing to the limited bandwidth (20 MHz) of the arbitrary function generator utilized. Furthermore, prior research has demonstrated that consideration of nonlinear contributions to the electro-optic effect is unnecessary up to a field strength of 65 kV/mm^30^. Hence, we conclude that deviations from linearity in frequency chirps within the experiment described above stem either from limitations in the evaluation algorithm or from equipment constraints. In essence, as long as the voltage supply provides a linearly increasing driving signal, the adiabatic frequency converter generates a perfectly linear frequency chirp.

Now, we want to discuss the coherence properties of the frequency shifted light. In our scenario, coherence length is determined by the linewidth of the laser, the resonance bandwidth and the noise of the resonance frequency. The linewidth of the laser is below 100 kHz, far below the resonance bandwidth of 1 MHz ($Q = 2\times 10^8$). The beat signals (second row in Fig. 2) were recorded with 20 kHz repetition rate over 1 hour, meaning we have switched between coupling light into the resonator and frequency shifting it for almost $10^8$ times without any observable signal distortion. Hence, it can be inferred that the noise of the resonance frequency over this hour-long period is considerably below 1 MHz. These results suggest that the coherence length exceeds 100 m and is primarily limited by the resonance bandwidth, specifically by the resonator’s decay time. Generally, the latter is dictated by the resonator loss. Recent studies have demonstrated that employing optical gain in resonators made of laser-active materials can substantially reduce this loss^31,32^. By utilizing such a scheme, it may be feasible to achieve even longer decay times and consequently longer coherence lengths.

With the generation of linear frequency chirps and an estimated coherence length of 100 m, we anticipate the capability to reliably determine distances in the 10-m range using FMCW LiDAR. The temporal evolution of the recorded beat frequency should exhibit a pattern akin to that depicted in Fig. 3b. Indeed, our experimental results displayed in Fig. 4a, nicely agree with this expectation.

At zero distance, we record a beat frequency of $f_{\textrm{B}}=60$ MHz with a rise time of $t_{\textrm{chirp}}=130$ ns. Given $B=690$ MHz, this corresponds to a time delay of $\Delta t=11$ ns. This delay arises because one of the arms of the fiber-based interferometer incorporates an additional 1.1 m long glass fiber with a refractive index of 1.5. This deliberate addition ensures a non-zero beat signal at zero distance, as illustrated in Fig. 6.

The distance resolution can be estimated as $c_0/(2B)\approx 22$ cm^6^. Comparing the peaks of the Fourier transform for distances of 535 cm and 555 cm suggests that our FMCW LiDAR system operates under bandwidth limitation, confirming the linearity of the frequency chirps. The measurement duration is constrained by the resonator’s decay time, approximately 100 ns. Consequently, the maximum total path difference is limited to 30 m, corresponding to 15 m target distance in air. We have successfully measured distances up to 10 m, being limited by the dimensions of our laboratory. In summary, electro-optically driven adiabatic frequency converters demonstrate excellent linearity in generating frequency chirps, with a proven coherence length exceeding 20 m. Moreover, these converters serve as suitable light sources for practical applications.

At first glance, the bandwidth of the chirp may not appear particularly impressive. Nevertheless, our study underscores the significant potential of electro-optically driven adiabatic frequency converters for ultrafast and practical continuous tuning of laser light. We have showcased a tuning range of 690 MHz in 65 ns, translating to an approximate tuning rate of 10 GHz/µs. These values position our systems to compete with the fastest tunable self-injection locking laser systems^18^. Notably, in our investigation, the deviation from perfect linearity is lower, primarily limited by the equipment rather than by the tuning process itself. Moreover, the remarkable potential of our tuning rate becomes even more apparent when considering the recently developed on-chip version of an electro-optically driven adiabatic frequency converter^26^. Here, tuning of 14 GHz was achieved with a 14-ps-long voltage step. Such a high tuning rate surpasses values attained with all other frequency tuning mechanisms by several orders of magnitude, including the improved version of self-injection locking^20^. Importantly, this potential is far from reaching its limit: Studies have demonstrated that lithium niobate can withstand electric fields of up to 65 kV/mm^30^ without exhibiting nonlinearity in the electro-optic response. This suggests the possibility of tuning the frequency of laser light by close to one terahertz. Consequently, achieving linear frequency tuning spanning several hundred gigahertz in less than a nanosecond, with coherence lengths of tens of meters, using a lithium niobate-based photonic integrated circuit, appears to be within reach.

It’s important to note that effective tuning can only occur if the voltage variation happens on a timescale significantly shorter than the decay time of light within the resonator. Achieving rise times beyond the microsecond range is challenging with lithium niobate^33^. Additionally, any measurement relying on adiabatic frequency tuning of laser light must be conducted within the cavity’s decay time, and it’s essential to acknowledge that the output power is not constant during this process.

Despite this limitation, our findings suggest that adiabatic frequency conversion presents a viable approach for continuously modulating the frequency of laser light by hundreds of gigahertz in sub-nanosecond timescales, exhibiting inherent tuning linearity and coherence lengths exceeding 10 m. To date, no other method for frequency tuning has achieved such capabilities. Moreover, we have demonstrated the suitability of adiabatic frequency converters for demanding applications like FMCW LiDAR, which necessitates high-performance light sources. However, the results from this study alone are insufficient to assert that adiabatic frequency converters surpass state-of-the-art light sources for FMCW LiDAR in all aspects. While AFC may offer the fastest linear frequency chirps, a fair comparison requires additional experiments, including assessments of velocity measurement and 3D mapping capabilities. Furthermore, one should demonstrate the ability to determine distances beyond 100 m.

However, various applications requiring ultrafast frequency tuning stand to benefit significantly from this tuning approach. Therefore, adiabatic frequency conversion emerges as a highly promising platform for demanding applications in trace gas measurements, which demand high acquisition rates and frequency tuning across different spectral regions^34^. While our research focuses on investigating continuous adiabatic frequency conversion for generating rapid frequency chirps, our findings suggest something more profound: the utilization of electro-optically driven frequency converters offers notable advantages over directly tuning the laser itself. There is no inherent competition between the lasing and conversion processes; the former does not constrain the latter, particularly in terms of tuning rate. Employing the linear electro-optic effect enables electrically controlled rapid, linear, and continuous tuning while maintaining a high level of coherence in the laser light.

## Materials and methods

### Resonator fabrication

**Figure 5:**
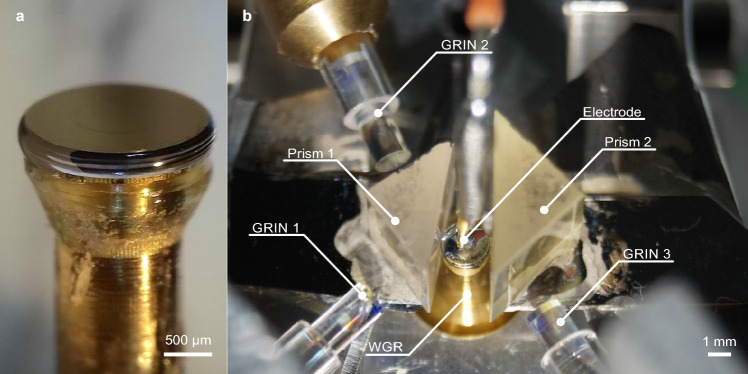
Photographs of key components for adiabatic frequency conversion. (**a**) Whispering gallery resonator with chromium electrodes on a brass post. (**b**) Experimental setup including the resonator (WGR), coupling prisms (Prism 1,2), GRIN lenses (GRIN 1,2,3) and electrode.

The resonator for this experiment was fabricated from a 300-µm-thick wafer of z-cut 5%-MgO-doped congruent lithium niobate. A 150-nm layer of chromium was deposited on both sides of the wafer. Subsequently, a cylindrical preform with a 3 mm diameter was shaped using a 388-nm wavelength femtosecond pulsed laser operating at a repetition rate of 2 kHz and an average output power of 300 mW. This preform was then soldered onto a brass post for further processing. Next, employing the same femtosecond pulsed laser in conjunction with a computer-controlled lathe, the rim of the resonator was shaped. Following the shaping procedure, the resonator measured 1.25 mm (major radius), 0.38 mm (minor radius), and 0.3 mm (thickness). To achieve a high-quality surface finish, the rim of the resonator was polished using a diamond slurry as fine as 50 nm. Finally, the resonator exhibited an intrinsic quality factor of $2\times 10^8$ at 1560 nm for extraordinary polarized light. Figure 5a depicts a photograph of the whispering gallery resonator.

### Linearity characterisation

Figure 1 sketches the experimental arrangement designed for characterizing the linearity of adiabatic frequency tuning. Additionally, Fig. 5b presents a photograph showcasing the key components. First, light from a laser diode emitting at 1560 nm wavelength and a typical linewidth of < 90 kHz is directed through a fiber. Extraordinarily polarized light is then focused by a gradient index lens (GRIN 1) and coupled into the resonator via a rutile prism (Prism 1). The distance between the prism and the resonator can be adjusted using a piezoelectric actuator. Moreover, the temperature of the resonator holder is stabilized at 28 ^∘^C, with residual fluctuations at the millikelvin level. Subsequently, GRIN lens 2 collects light from the output facet of Prism 1, comprising both the light reflected at the prism base and the light coupled out of the resonator. The latter undergoes frequency modulation due to adiabatic frequency conversion. Consequently, the interference between these two light fields generates a beat signal, which is then recorded.

To characterize linearity, we apply a voltage that steadily increases from 0 to 20 V (as depicted in the top row of Fig. 2), resulting in a corresponding temporal variation in the frequency of the intracavity light. We assume that the frequency of the pump light remains constant throughout the frequency tuning process, which is reasonable for tuning times in the range of 100 ns. Under this assumption, the beat frequency corresponds to the adiabatically-induced frequency shift. To extract the beat frequency, i.e. the frequency shift, we analyze the recorded signal (shown in the second row of Fig. 2) using a short-time Fourier transform. This method, utilized for the same purpose, was previously employed by Snigirev et al.^18^, with documentation and a Python code available in Ref.^35^. We implemented this analysis using Matlab, and the corresponding code along with raw data are provided in Ref.^36^.

The temporally varying frequency shift is displayed in the third row in Fig. 2. In order to quantify the linearity, we subtract a perfect linear frequency increase (bottom row in Fig. 2).

### Coherent distance measurement experiment

For the coherent ranging experiment, the frequency modulated light is coupled out via prism 2 and collected using GRIN lens 3 (see Fig. 5b). Its power is amplified by an erbium-doped fiber amplifier (EDFA) from 10 µW to 14 mW, following which it is devided into two paths. 10 % of the power serves as the reference signal, directed towards a balanced photo diode. 90 % of the power is directed towards the target, which in this case is a non-polished metal plate, via a circulator and a collimator (7.5 mm focal length, numerical aperture $NA = 0.3$). The light reflected from the target is collected with the collimator and directed to the balanced photodiode, where it interferes with the reference light. Due to the path difference, these two light fields form a temporally varying beat signal which is recorded.

**Figure 6:**
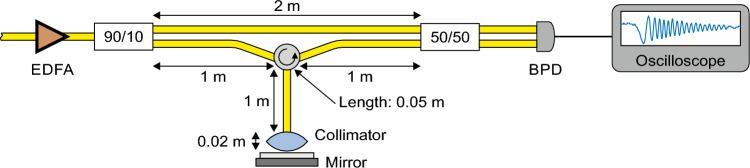
Sketch of the experimental setup for the zero distance measurement. The additional 1.1-m-long glass fiber in one of the fiber-based interferometer arms ensures a non-zero beat signal at zero distance. To measure the frequency at zero distance, we placed a mirror directly in front of the collimator, resulting in a measured beat signal of $f_{\textrm{B}}=60$ MHz at $t_{\textrm{chirp}}=130$ ns rise time. This corresponds to $\Delta t=11$ ns time delay.

To ascertain the beat frequency $f_{\textrm{B}}$ from the recorded signal, we employ the same methodology as used for the linearity analysis (section “Linearity characterisation”). Assuming linear frequency modulation, the maximum beat frequency should be directly proportional to the distance between the collimator and the target:

2 $$\begin{aligned} f_{\textrm{B}}=\frac{B}{t_{\textrm{chirp}}}\Delta t =\frac{B}{t_{\textrm{chirp}}}2\left( \frac{\Delta D_{\textrm{f}} \, n_{\textrm{f}}}{c_0}+\frac{D}{c_0}\right) \;, \end{aligned}$$

with the chirp bandwidth *B*, the chirp time $t_{\textrm{chirp}}$. The difference $\Delta D_{\textrm{f}}$ of the fiber lengths between reference and target arms and the refractive index $n_{\textrm{f}}$ of the fibers and the distance *D* contribute to the time delay $\Delta t$ between reference light and target light reflected by the metal plate. In our experiment, we have $\Delta D_{\textrm{f}}=1.1$ m and $n_{\textrm{f}}\approx 1.49$. Thus, we expect a linear relation between beat frequency $f_{\textrm{B}}$ and distance *D* having 60 MHz offset at zero distance. We confirm this offset by putting a mirror only 2 cm away from the collimator (Fig. 6).

## Data Availability

The raw data along with the source code for data analysis used in this work are available on the Zenodo repository^36^.
